# Discontinuation and dose reduction of rituximab in relapsing–remitting multiple sclerosis

**DOI:** 10.1007/s00415-021-10399-8

**Published:** 2021-01-21

**Authors:** Malin Boremalm, Peter Sundström, Jonatan Salzer

**Affiliations:** Department of Clinical Sciences, Neurosciences, Umeå University, Umea, Sweden

**Keywords:** Rituximab, Relapsing–remitting multiple sclerosis, Observational study, Dose reduction

## Abstract

**Background:**

Rituximab is safe and effective for treating relapsing–remitting multiple sclerosis (RRMS) according to phase II and observational studies. There are limited data on disease activity after discontinuation and dose reduction. The objective of this study was to evaluate the effects on inflammatory disease activity after discontinuation or dose reduction of rituximab in patients with RRMS or clinically isolated syndrome (CIS).

**Methods:**

In this retrospective observational study, we included all RRMS and CIS patients ever treated with rituximab at the University Hospital of Umeå who had either; (1) discontinued treatment at any time or (2) reduced the dose to a mean of < 1000 mg yearly. The patients served as their own controls by contributing patient years on full dose, reduced dose, and off treatment.

**Results:**

A total of 225 patients treated with mean (SD) 6256 (2456) mg rituximab during mean (SD) 6.5 (2.0) years were included. There were no differences regarding the annualized relapse rates during full dose versus reduced dose or off treatment (0.02 versus < 0.01 and 0.02, *p* = 0.09), neither regarding proportion MRI scans with new or enlarged T2 lesions (0.03 versus 0.01 and 0.03, *p* = 0.37) or contrast-enhancing lesions (< 0.01 versus 0 and 0.02, *p* = 0.22).

**Conclusions:**

This study indicates that rituximab has long-term effects on inflammatory disease activity and that disease reactivation is rare in MS patients who discontinued treatment for any reason. It also suggests that treatment with low-dose rituximab (< 1000 mg yearly) is sufficient to maintain suppression of inflammatory disease activity in patients with stable disease.

## Introduction

In relapsing–remitting multiple sclerosis (RRMS), inflammatory disease activity decreases with higher age and longer disease duration [1–3]. The currently available disease-modifying treatments are aimed at suppressing inflammatory activity [4], and younger age and higher disease activity are associated with larger relative treatment benefits [5]. In a 48 weeks long phase II study, the monoclonal anti-CD20 B-cell depleting antibody rituximab at a dose of 1000 mg given intravenously day 1 and 15 was superior to placebo in suppressing inflammatory disease activity for patients with RRMS [6]. Several retrospective observational studies also show that rituximab is a safe and highly effective treatment option with a low drug discontinuation rate [7–11]. At the University Hospital of Umeå, in northern Sweden, > 90% of all patients with RRMS are treated with rituximab. The most common treatment regimen is one infusion with 1000 mg of rituximab as soon as possible after disease onset, then 500 mg every 6 months for 3 years, followed by dose reduction to 500 mg yearly, provided a stable disease course [12]. Some patients receive a lower dose, have extended dosing intervals or discontinue treatment for different reasons such as stable disease course, age, pregnancy, adverse effects etc. However, there are limited data on disease activity after dose reduction and discontinuation of treatment with rituximab.

## Methods

### Study population

**Fig. 1:**
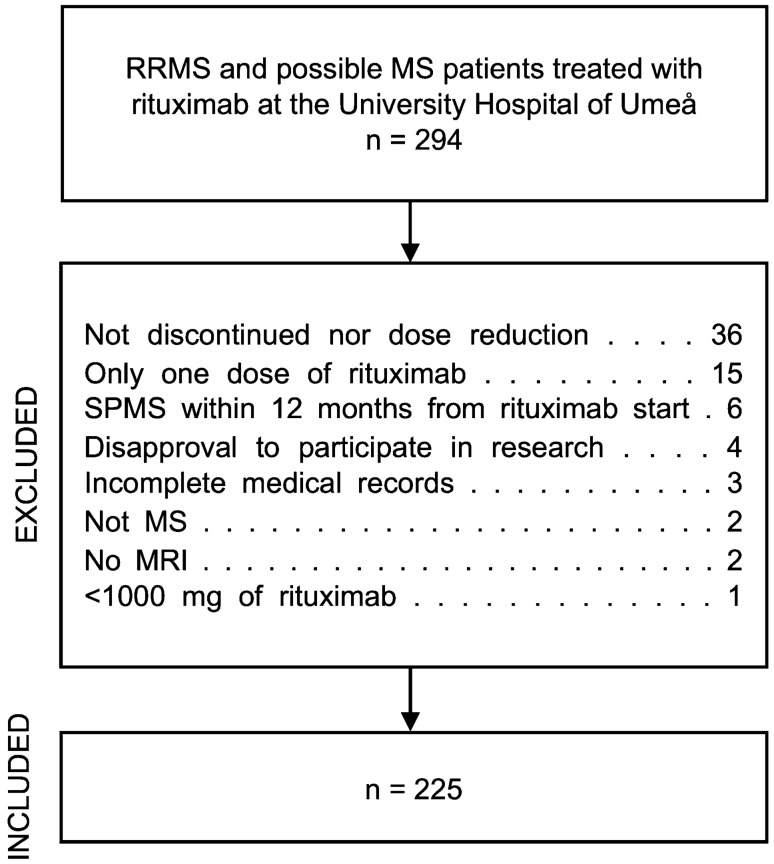
Flowchart depicting the selection process of the 225 patients constituting the study cohort. An extraction was made from the Swedish multiple sclerosis (MS) registry on 7 May 2020 selecting patients with relapsing–remitting MS (RRMS) or CIS ever treated with rituximab at the University hospital of Umeå. Inclusion criteria were all RRMS and CIS patients ever treated with rituximab who either; (1) had discontinued treatment at any time (i.e. ≥ 18 months since last infusion) or (2) had reduced the treatment dose to a mean of < 1000 mg rituximab yearly. Exclusion criteria were **a** patients who had transitioned to secondary progressive MS (SPMS) within 12 months from starting rituximab treatment, **b** patients who only received one dose of rituximab, **c** patients who received a total of < 1000 mg of rituximab, **d** incomplete medical records, **e** no MRI examinations during the observation period or **f** disapproval to participate in research

Figure 1 depicts the selection process of the 225 patients constituting the final study cohort. The source population was all patients with multiple sclerosis or clinically isolated syndrome (CIS) with a high risk for transitioning to MS (i.e., patients with one relapse fulfilling MRI criteria for dissemination in space but not time) in the Swedish MS registry (http://www.neuroreg.se) at the University Hospital of Umeå. The registry data extraction was performed 7 May 2020, selecting all RRMS or CIS patients ever treated with rituximab at the University Hospital of Umeå. Patients were included if they fulfilled any of the following inclusion criteria; either (a) had discontinued treatment with rituximab at any time (i.e. ≥ 18 months since last infusion) or (b) reduced the treatment dose to a mean of < 1000 mg of rituximab yearly. Exclusion criteria were: (a) patients who was diagnosed with secondary progressive MS (SPMS) within 12 months from starting rituximab treatment, (b) patients who had only received one dose of rituximab, (c) patients who had received a total of < 1000 mg of rituximab, (d) incomplete medical records, (e) no MRI examinations during the observation period or (f) disapproval to participate in research.

The study was approved by the local ethics committee in Umeå (2013/445-31) and all patients had provided oral consent to participate in the MS registry.

### Data collection

Data were extracted from the Swedish MS registry and patients’ medical records and included age, sex, diagnosis and disease course, date of disease onset, Expanded Disability Status Scale (EDSS) scores, dates and doses of rituximab infusions, dates of MRI examinations, numbers of T2 lesions and CEL(s) on MRI and dates of relapses. The baseline EDSS score and baseline MRI were defined as the examination closest in time within 12 months prior to starting rituximab. For patients lacking an EDSS score or an MRI within this timeframe, the baseline value was obtained from within 3 months after starting rituximab, if available. Date of data censure was 7 May 2020. If a patient had received any other disease-modifying therapy (DMT) during the observation period, the time after administration of that drug was excluded from further analyses. If a patient had converted to SPMS, the year of conversion was noted in the registry. If this had occurred during the observation period, all time from 1 January that year was excluded from further analyses. All patients were followed from 3 months after the first rituximab infusion until the date of data censure or lost to follow-up (conversion to SPMS, started another DMT, migration or absent from clinical follow-up), whichever came first.

### Predictors and outcomes

**Fig. 2:**
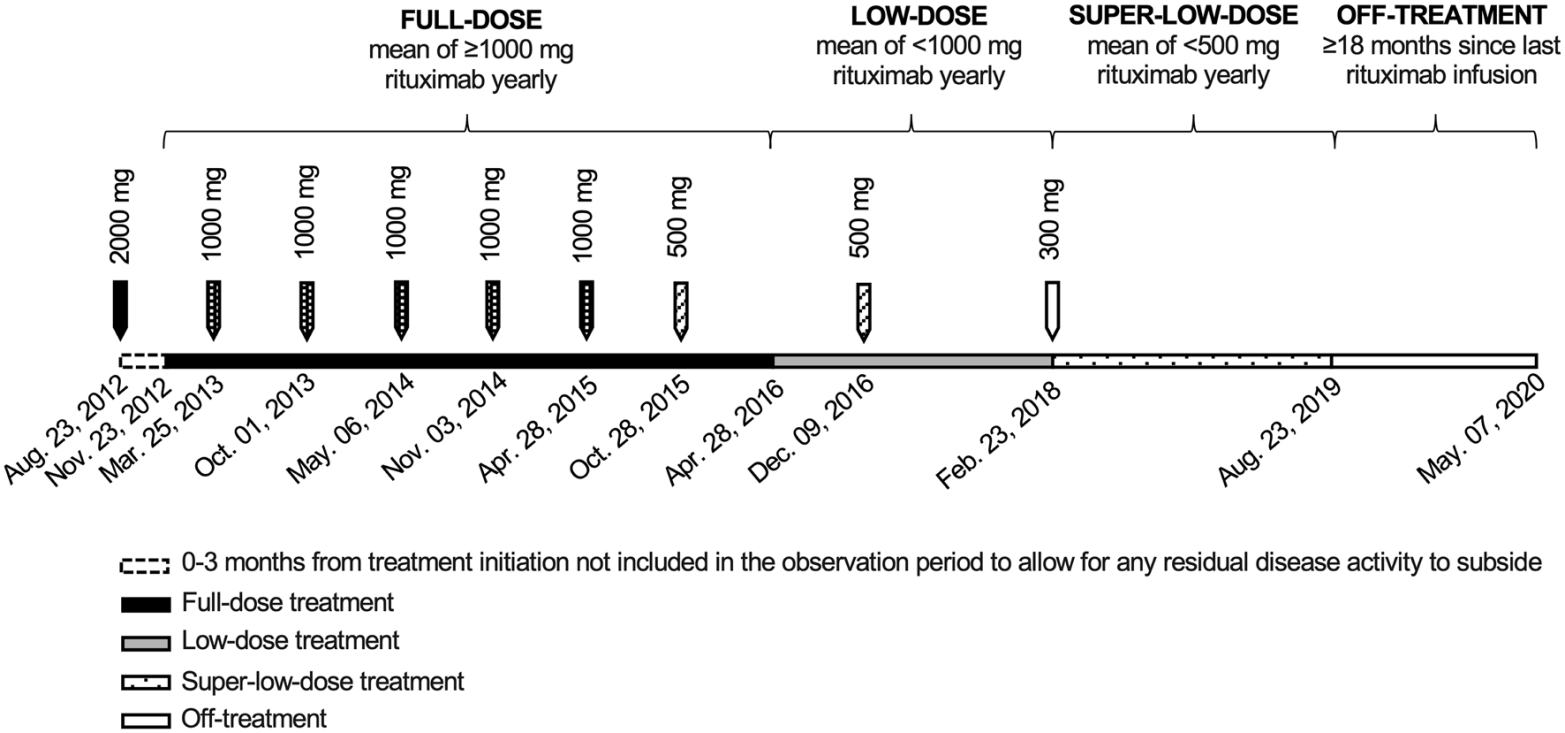
Illustration of a patient case with doses of rituximab, dates of infusions and the different treatment periods over time

Inflammatory disease activity was defined as either relapse, new or enlarged T2 lesions and/or CEL(s) on MRI. Concerning CEL(s), a valid scan was defined as an MRI performed with gadolinium contrast administration, and a positive scan was defined as such an MRI with CEL(s). A clinical relapse was defined as an episode of neurological impairment with a duration of > 24 h without another apparent cause. The patients served as their own controls by contributing with patient years on both full dose, low dose, super-low dose and/or off treatment during the observation period (Fig. 2). The definition for time on full-dose treatment was the period during which the mean dose of rituximab was ≥ 1000 mg yearly. The full-dose observation period started at 3 months after initiating treatment with rituximab, or at 3 months after the first re-start infusion of rituximab following a period off treatment, to allow for any residual disease activity to subside. The definition for time on low-dose and super-low-dose treatments was the time during which the mean yearly rituximab dose was < 1000 mg and < 500 mg, respectively. In cases where the dosing interval with 500 mg of rituximab was extended from every 6 to every 12 months, the transition from full dose to low dose was considered to happen 6 months after the last 500 mg infusion. In cases where the infusions with 1000 mg of rituximab was given every 12 months, the transition from full-dose to low-dose was considered to happen at the date of the first infusion of 500 mg. The same definitions applied to the transition from full dose to super-low dose with the difference that the yearly dose of rituximab was reduced to < 500 mg. The effect duration of one rituximab infusion was approximated to 18 months [13] and the definition for time off treatment was the time that exceeded 18 months after an infusion. Disease activity was allocated to the off-treatment group if it appeared after 18 months since last rituximab infusion or within 3 months of rituximab restart at any time after 18 months untreated.

### Statistical analyses

Statistical analyses were performed using IBM SPSS Statistics for Windows, Version 26.0 (Armonk, NY: IBM Corp). Normally distributed data were presented as mean (SD) and non-normally distributed data were presented as median (IQR). The independent samples *t* test was used to compare means and the Mann–Whitney *U* test was used to compare medians. Proportions were compared with the Pearson *X*^2^ test, and if one category had less than five observations the Fisher’s exact test was used. The ARR and the proportion of MRI scans with new or enlarged T2 lesions and CEL(s) was compared using the Fisher’s exact test in Win-pepi software version 11.65.

## Results

### Study population

Table 1 presents the baseline characteristics of the 225 patients who were included in the final study cohort (of which 17 patients with CIS). A large proportion (46.7%) of the patients had a high lesion burden of > 20 T2 lesions and a quarter (24.9%) of the patients had signs of active disease with CEL(s) at baseline. Eleven patients were lost to follow-up before date of data censure; four patients converted to SPMS, five patients received treatment with another DMT, one patient migrated and one patient had been absent from clinical follow-up of unknown cause. Of the patients lost to follow-up (*n* = 11); one patient had 1 new T2 lesion on full-dose treatment and one patient had 1 new T2 lesion after discontinuation. The remaining nine patients had no disease activity on any dose regimen before lost to follow-up. The baseline EDSS for the study cohort was assessed at a median of 14 (2–43) days prior to the first rituximab infusion and the baseline MRI was performed at a median of 26 (7–71) days prior to rituximab start, excluding those with MRI and EDSS assessments performed after rituximab initiation.

**Table 1:** Baseline characteristics of 225 patients with relapsing remitting multiple sclerosis (MS) or possible MS treated with rituximab who either had reduced the dose to a mean of < 1000 mg yearly and/or discontinued treatment at any time

Characteristics	*n* = 225
Age, years, mean (SD)	38.4 (11.2)
Female sex, *n* (%)	162 (72.0)
MS duration, years, median (IQR)	5.8 (1.6–11.3)
Possible MS, *n* (%)	18 (8.0)
Baseline EDSS score^a^, median (IQR)	2.0 (1.0–2.5)
Number of T2 lesions on baseline MRI^a^, *n* (%)	
0	2 (0.9)
1–9	52 (23.1)
10–20	61 (27.1)
> 20	105 (46.7)
CEL(s) on baseline MRI, *n* (%)	56 (24.9)
Follow-up time total, years, mean (SD)	6.5 (2.0)

*SD* standard deviation, *IQR* interquartile range, *MS* multiple sclerosis, *EDSS* expanded disability status scale, *MRI* magnetic resonance imaging, *CEL(s)* contrast enhancing lesion(s)

^a^Baseline EDSS score and baseline MRI were defined as the examination closest in time within 12 months prior to starting rituximab. For patients lacking an EDSS score or an MRI within this timeframe, the baseline score was obtained from within 3 months after starting rituximab, if available (EDSS score, eight patients; MRI, one patient). Missing data: baseline EDSS, seven patients; number of T2 lesions on baseline MRI, five patients; CELs on baseline MRI, 11 patients

### Outcomes

The distribution of outcomes by treatment period is seen in Table 2 with a total of 1457.8 patient-years at risk. During the observation period 191 patients reduced the dose to < 1000 mg yearly, 36 patients reduced the dose to ≤ 500 mg yearly and 111 patients discontinued treatment. The frequency of MRI scans with gadolinium contrast per patient year was lower during the low-dose and super-low-dose treatment periods with 0.3 and 0.2 yearly MRI scans, respectively, compared to 1.0 and 0.7 MRI scans with gadolinium contrast per year for time on full dose and off treatment. There were no significant differences in proportions of MRI scans with new or enhanced T2 lesions or proportion of MRI scans with CEL(s) between the treatment periods. There were a total of 25 relapses during the observation period; 20 on full dose, two on low dose, and three relapses off treatment. The ARRs were low and no significant difference was seen between the different treatment periods. One hundred and eleven patients (49.3%) had discontinued treatment with rituximab at some point during the observation period and thus contributed to patient years at risk during off treatment. Seventy-six patients had discontinued treatment once, 31 patients twice; and four patients had three separate time periods off-treatment. The most common reason for discontinuation was adherence to a study protocol inferring discontinuation of rituximab after an accumulated dose of 2000 mg (36.0%) and the second most common reason was stable disease course (24.7%) (Table 3). Fifty-three patients were off treatment at the date of data censure.

**Table 2:** Distribution of outcomes by treatment period: full dose (mean of ≥ 1000 mg rituximab yearly), low dose (mean of < 1000 mg rituximab yearly), super-low dose (mean of < 500 mg rituximab yearly) and off treatment (i.e. > 18 months after last infusion of rituximab)

Outcome	Full dose	Low dose	Super-low dose	Off treatment	*p* value
Patient years at risk	845.6	402.6	44.6	165.0	
Yearly dose rituximab, mg, median (IQR)	1413 (1203–1684)	433 (352–512)	303 (201–558)	N/A	
Yearly dose rituximab, mg, mean (SD)	1438 (321)	446 (215)	269 (1208)	N/A	
T2 lesions					
Number of positive scans	28	5	1	5	
Number of valid scans^a^	928	362	41	172	
Number of positive scans/number of valid scans	0.03	0.01	0.02	0.03	0.37
MRI per patient year	1.1	0.9	0.9	1.0	
CEL(s)					
Number of positive scans	4	0	0	2	
Number of valid scans^b^	884	135	9	112	
Number of positive scans/number of valid scans	< 0.01	0	0	0.02	0.22
MRI with gadolinium contrast per patient year	1.0	0.3	0.2	0.7	
Relapse					
Number of relapses	20	2	0	3	
Annualized relapse rate	0.02	< 0.01	0	0.02	0.09

*IQR* interquartile range, *SD* standard deviation, *N/A* not applicable, *MRI* magnetic resonance imaging, *CEL(s)* contrast enhancing lesions

^a^MRI with or without gadolinium contrast

^b^MRI with gadolinium contrast

**Table 3:** Reasons for discontinuation of rituximab for the 150 time periods off treatment (i.e. > 18 months since last infusion) contributed by 111 patients during the observation period

Reason for discontinuation	*n* (%)
Study participation^a^	54 (36.0)
Stable disease	37 (24.7)
Low levels of IgG	16 (10.7)
Dosing interval of > 18 months	16 (10.7)
Susceptibility to infections	10 (6.7)
Pregnancy	8 (5.3)
Adverse events	7 (4.7)
Infection	1 (0.7)
Ongoing disease preventing treatment	1 (0.7)
Total number of time periods off treatment	150

^a^Adherence to a study protocol inferring discontinuation of rituximab after an accumulated dose of 2000 mg

We compared the group of patients who regained disease activity (*n* = 15) with those who did not (*n* = 210) after dose reduction or discontinuation of rituximab (Table 4). More (33% versus 14%) of the patients with disease activity had experienced breakthrough disease on full-dose treatment and they also had a higher baseline EDSS (*p* = 0.01) compared with the group with no disease activity. The latest available EDSS score during the observation period was assessed at a mean of 8.1 (1.9) and 6.0 (2.0) years from baseline for patients with disease activity and patients with no disease activity, respectively. Minimal changes in EDSS (− 0.5 in both groups) were observed during the observation time.

**Table 4:** Characteristics of 225 patients treated with rituximab compared by groups of patients who regained disease activity compared to patients who did not regain disease activity after dose reduction (to < 1000 mg of rituximab yearly) or discontinuation (i.e. > 18 months since last infusion) of rituximab

Characteristics	Disease activity (*n* = 15)	No disease activity (*n* = 210)	*p* value
Female sex, *n* (%)	11 (73.3)	151 (71.9)	1.00
Age at first rituximab infusion, years, mean (SD)	35.5 (15.0)	38.6 (10.9)	0.31
MS duration at first rituximab infusion, years, median (IQR)	3.5 (1.2–11.6)	5.8 (1.6–11.3)	0.86
Baseline EDSS score, median (IQR)	2.5 (1.8–3.5)	2.0 (1.0–2.5)	0.01
Number of T2 lesions on baseline MRI, *n* (%)			
0	1 (6.7)	1 (0.5)	N/A
1–9	1 (6.7)	51 (24.3)	N/A
10–20	5 (33.3)	56 (26.7)	N/A
> 20	8 (53.3)	97 (46.2)	N/A
CEL(s) on baseline MRI, *n* (%)	4 (26.7)	52 (24.8)	0.84
Accumulated dose on full-dose treatment, mg, mean (SD)	4753 (3144)	5355 (2169)	0.32
Relapse on full-dose treatment, *n* (%)	3 (20.0)	15 (7.1)	0.11
New or enlarged T2 lesions on full-dose treatment, *n* (%)	4 (26.7)	18 (8.6)	0.05
CEL(s) on full-dose treatment, *n* (%)	0 (0.0)	2 (1.0)	1.00
Any disease activity on full-dose treatment^a^, *n* (%)	5 (33.3)	30 (14.3)	0.06
Age at disease activity, years, mean (SD)	40.3 (15.0)	N/A	N/A
MS duration at disease activity, years, median (IQR)	11.4 (5.3–16.1)	N/A	N/A
Time from first rituximab infusion to disease activity, years, mean (SD)	4.8 (2.7)	N/A	N/A

*N/A* not applicable, *SD* standard deviation, *IQR* interquartile range, *MS* multiple sclerosis, *EDSS* expanded disability status scale, *MRI* magnetic resonance imaging, *CEL(s)* contrast enhancing lesion(s)

^a^Defined as either relapse or new or enhanced T2 lesion and/or CEL(s) on MRI. Missing values disease activity: baseline EDSS score, one patient; new or enlarged T2 lesions on full-dose treatment, one patient; CEL(s) on full-dose treatment, two patients. Missing values no disease activity: baseline EDSS score, six patients; number of T2 lesions on basline MRI, five patients; CEL(s) on baseline MRI, 11 patients; new or enlarged T2 lesions on full-dose treatment, two patients; CEL(s) on full-dose treatment, two patients

Among the 15 participants with signs of disease activity after dose-reduction or discontinuation seven were on low dose, one were on super-low dose and seven patients were off treatment at the time of their first episode of disease activity. The reasons for dose reduction in seven of these patients were stable disease, which meant a dose reduction to 500 mg yearly according to the regular treatment protocol at the clinic, and low IgG levels for the patient on super-low-dose treatment. The reasons for discontinuing treatment with rituximab in the remaining seven patients with signs of disease activity after discontinuation were pregnancy (*n* = 1), stable disease (*n* = 1), dosing interval of 24 months (*n* = 1) and following a clinical study protocol [13], which meant stopping treatment after two doses of 1000 mg of rituximab (*n* = 4). Among the 15 patients showing signs of disease activity 11 had new or enlarged T2 lesion(s) on MRI (1–2 lesions); two of these patients also had one CEL (both patients off-treatment following a study protocol [13]) and four patients experienced a relapse (two after dose-reduction and two after discontinuation).

## Discussion

In this retrospective observational study on patients with RRMS or CIS, we evaluated the effects on inflammatory disease activity after discontinuation and dose reduction (to a mean of < 1000 mg yearly) of rituximab treatment. The results indicate that rituximab has long-term effects on inflammatory disease activity and that disease reactivation is rare in previously rituximab treated RRMS and CIS patients who discontinued treatment. Our study also implies that a low-dose maintenance treatment protocol may be sufficient for RRMS and CIS patients with stable disease.

Patients who had transitioned to SPMS within 12 months from starting rituximab were excluded from this study as clinicians tend to be cautious and frequently diagnose SPMS retrospectively after a period of diagnostic uncertainty, resulting in a delay of SPMS diagnosis by up to 3 years [14].

A high proportion of patients had a heavy T2 lesion burden and CEL(s) at baseline which suggests that our study population was inflammatory active at inclusion. Our results confirm a low ARR and a low risk for new lesions on MRI during treatment with rituximab, in line with previous studies [6–11, 13]. The two patients with CEL(s) on MRI after dose reduction or discontinuation both discontinued treatment within the frame of a clinical study and had only received two rituximab infusions (1000 mg i.v. days 1 and 15 according to study protocol) before stopping treatment [13]. In the standard clinical setting at the University Hospital of Umeå these patients would have continued full-dose treatment for at least 3 years before considering dose reduction, provided a well-tolerated treatment and a stable disease course. There is unfortunately no clear definition of “stable disease course”; however as a rule of thumb patients without signs of clinical or radiological disease activity (i.e. no relapses, stable EDSS, no new or contrast enhancing lesions on yearly MRIs) during the last 3 years are considered for dose reduction. During the 400 patient years on low-dose treatment in this study, the frequency of disease breakthrough was low which indicates that treatment with a low-dose protocol (i.e. < 1000 mg yearly) is sufficient to maintain suppression of inflammatory disease activity in patients with RRMS and CIS with stable disease.

Pregnancy is one of the most common reasons for discontinuing rituximab [9, 15] and since the late 1990s, it is well known that there are fewer MS relapses during pregnancy, especially in the later trimesters, and that the risk of relapse increases in the early postpartum period, especially in women with active disease before pregnancy [16]. However, in a more recent study on a contemporary MS cohort with patients diagnosed earlier due to new diagnostic criteria, no increased disease activity was seen postpartum [17]. As for MS drugs with shorter effect duration than rituximab the risk management during washout periods before conception needs to be addressed, since these are associated with a risk of rebound disease activity [18–20]. Our findings of a large proportion of patients without any sign of disease reactivation after discontinuation of rituximab suggests that inflammatory disease activity stays suppressed long after cessation of treatment. These findings corroborate the notion that part of the effect of B-cell depleting drugs is mediated through memory B-cell depletion [21–23]. This long-term disease suppression seems to be able to mitigate the increased risk of disease activity associated with a pregnancy for women with highly active disease, making B-cell depletion an attractive treatment option in fertile females [24].

In the natural history of MS, the inflammatory activity is at its highest early in the disease and generally decreases with age and disease duration [2, 3]. Younger age and higher disease activity are also associated with larger relative treatment benefits [5]. These observations, together with the results of the current study, suggest that full-dose treatment with rituximab at disease onset with gradual tapering of treatment intensity may be a successful treatment strategy with retained suppression of disease activity.

A few patients had a very short observation time on low- and super-low dose which results in a misleading dispersion of the yearly rituximab dose (Table 2). The frequency of MRI scans with gadolinium contrast during low-dose and super-low-dose treatment was lower compared with during full-dose treatment. This is explained by the fact that the MRI follow-up protocol in Sweden stipulates that after approximately 3 years of stable disease without signs of disease activity gadolinium is no longer routinely administered. The main limitation of this study is its inherent shortcomings based on its retrospective, non-randomized design. Larger randomized studies are needed to gain higher levels evidence regarding optimal rituximab doses, treatment intervals, and treatment duration in RRMS patients. The short observation time after dose reduction and discontinuation in the current study prevents conclusions regarding longer term effects of disease activity. The ongoing prospective randomized phase 3 study RIDOSE-MS, comparing a 12-month dosing interval of 500 mg rituximab with a 6-month dosing interval, may potentially provide data which allow more firm conclusions to be drawn [25].

## Conclusion

This study indicates that rituximab has long-term effects on inflammatory disease activity and that disease reactivation is rare in previously rituximab treated RRMS and CIS patients who discontinued treatment for any reason. It also suggests that treatment with a low-dose protocol of rituximab (< 1000 mg yearly) is sufficient to maintain suppression of inflammatory disease activity in most RRMS and CIS patients with stable disease. These findings could be useful to aid treatment decisions for RRMS patients during the COVID-19 pandemic during which the risk/benefit ratio of immunosuppressive treatment for MS may shift.

## Data Availability

Data available on request from the authors.
